# COP-eration for global food security

**DOI:** 10.12688/f1000research.10323.1

**Published:** 2016-12-05

**Authors:** Erick de la Barrera

**Affiliations:** 1 Instituto de Investigaciones en Ecosistemas y Sustentabilidad, Universidad Nacional Autónoma de México, Morelia, Michoacán 58190, Mexico

**Keywords:** Biodiversity, COP-13, food justice, global environmental change, human development, planetary boundaries, sustainability

## Abstract

Mexico is hosting the 13th Conference of the Parts (COP-13) on the Convention on Biological Diversity. Participants will have another opportunity to "integrate biodiversity for wellbeing." Considering that food production is a major driver for the loss of biological diversity, despite the fact that ample genetic reservoirs are crucial for the persistence of agriculture in a changing world, food can be a conduit for bringing biodiversity into people's minds and government agendas. If this generation is going to "live in harmony with nature," as the Aichi Biodiversity Targets indicate, such an integration needs to be developed between the agricultural and environmental sectors throughout the world, especially as an increasingly urban civilization severs its cultural connections to food origin.

The world is witnessing an accelerated loss of biological species owing to human activities, which are also putting the integrity of other life-support systems of the planet at risk ^1, 2^. In response, the governments of 196 countries (86% have signed thus far) established the Convention on Biological Diversity (CBD; http://www.cbd.int/) in 1992 with the objectives of 1) conserving existing biodiversity, 2) utilizing the components of biodiversity in a sustainable manner, and 3) ensuring that the benefits stemming from the use of genetic resources are distributed in a fair and equitable manner. Given concerns about the development of new biotechnologies, a series of provisions based on the cautionary principle have also been established in the Cartagena Protocol for Biosecurity ( https://bch.cbd.int/protocol/), which became valid in 2003. In addition, given the disparities between economic gains reaped by large multinational corporations and the conditions facing first nations, who are regarded as the custodians of biodiversity, the Nagoya Protocol became effective in 2014 to regulate access to genetic diversity and the sharing of its benefits ( https://www.cbd.int/abs/).

Cancún is host to the 13th Conference of the Parts (COP-13; 28 ^th^ November - 17 ^th^ December 2016) on the CBD with the guiding theme of "integrating biodiversity for wellbeing." This is no simple task, especially when considering that our western civilization relies on a conceptual separation of humans and nature. Perhaps food can be a useful conduit for incorporating the notion of biodiversity into everyday life. Indeed, the link between ecosystem integrity and food production is deceivingly strong. On one hand, agro-food production is a main driver for global environmental change. For instance, advancing the agricultural frontier results in the loss of natural ecosystems in agricultural exporter regions, such as the Brazilian Amazon for production of cereals, soybean, and cattle husbandry, the Argentine Pampas for production of soybean, and the Mexican cloud forests for the production of avocados. In addition, over 10% of the global greenhouse gas emissions stem from agriculture, including from the production of synthetic fertilizer and other agrochemicals, the operation of machinery, such as water pumps and tractors, and transporting inputs and produce to and from the sites of final consumption ^3^. On the other hand, agriculture is also one of the most vulnerable sectors to environmental change. A recent example is the multi-year drought in California, where more than 2 billion dollars and almost 20,000 jobs had been lost by 2014, in what otherwise was the most important agricultural state in the USA ^4^. Also, the production of coffee, the most traded global commodity after hydrocarbons, is likely to decrease during the present century, especially at lower elevations ^5^.

Food supply inherently relies on biodiversity. For instance, an ample genetic diversity for cultivated species and their wild relatives is necessary for the selection of materials with desirable traits for the development of improved varieties. Thus, the reservoirs of genetic diversity, along with traditional cultural practices, need to be protected, including for *Coffea arabica* in Ethiopia, *Zea mays* in the American continent, and *Oryza sativa* throughout Asia ^6– 8^. Also, a high diversity of edible species has originated from the prevalence of family agriculture, already responsible for 80% of the global food production ( http://www.fao.org/3/b-mm296e.pdf). It is precisely in home gardens throughout the world where domestication has occurred for numerous species that have been incorporated to local menus and pharmacopeias over the course of history. Finally, these catalogues of species will allow the development of new "climate-ready" crops, such as the cultivation of agaves for production of fiber, sugars, and ethanol in arid lands, reduction of methane emissions during rice cultivation systems, and bringing to mainstream agriculture new fruits, sources of starch, and vegetables, as our diet shifts to a less meat-intensive food system ^9– 11^.

However, people are increasingly disconnected from their food's origin. This is the result of multiple factors, including an increasingly urban population, the consequent rapid growth in the demand for food with a long shelf life, and the fact that large-scale cattle husbandry and industrialized foods rely on a mere handful of species. This is especially true for the urban poor, who only have access to highly processed food from convenience stores, and, to a lesser extent, for the middle classes, for whom eating out is aspirational and a parameter considered when quantifying wealth ( http://documents.worldbank.org/curated/en/269121467991958460).

It thus seems paradoxical that the prevalent government model has ministries of environment and agriculture as discrete entities, the environmental sector often regarded as obstructive to economic development. Examples include exceptions of environmental compliance for agricultural activities in the USA ^12^, the recent reopening of fisheries within an exclusion zone adjacent to oil platforms off the coast of Campeche in the Gulf of Mexico ( http://dof.gob.mx/nota_detalle.php?codigo=5456197&fecha=11/10/2016), and the internationally pervasive disconnection between government investment in the agricultural sector and a meager to nil improvement in food security and food related health issues ^13^. This antagonism makes sense from a historical perspective, though. After all, agriculture is the activity that allowed the rise of humans as Earth's dominant species. In contrast, our understanding of planetary life support systems only developed during the last century. Now we know that nature is not an external entity created for human utilization, but that the evolutionary and ecological success and the (sometimes debatable) cognitive awareness that characterize our species come with the responsibility of looking after the planet. An example of this paradigm shift is *Laudato si'* ( http://w2.vatican.va/content/francesco/en/encyclicals/documents/papa-francesco_20150524_enciclica-laudato-si.html), the papal encyclical whose second chapter argues against the literal interpretation of the so-called creation mandate that has justified the human (ab)use of nature over the course of many centuries.

The most important task of the CBD is perhaps to bring biological diversity to center stage in the minds of citizens and government agendas in a similar way to what the Framework Convention on Climate Change achieved after 23 years of existence ( http://unfccc.int/paris_agreement/items/9485.php). Indeed, the link between the optical properties of certain gases and ongoing changes in temperature and precipitation with the economy and the planet's viability is now considered to be straight forward. While the link between biological diversity and planetary viability appears to be subtler, monitoring its integrity can be done by direct observation so it can involve the public, a necessary step if we are to transit towards the Aichi Biodiversity Targets of living in harmony with nature. The food nexus may be an initial stepping stone.
