# Supplementary material for: COP-eration for global food security
Source: F1000Res. 2016 Dec 5;5:2814. [Version 1] doi: 10.12688/f1000research.10323.1 (PMC5225408; doi:10.12688/f1000research.10323.1)
Supplement: Peer review history

# Peer review: Referee response for version 1

**DOI:** 10.5256/f1000research.11119.r18233

**Published:** 2017-01-03

**Authors:** Enrico A. Yépez

**Affiliations:** 1 Department of Water and Environmental Sciences, Sonora Institute of Technology (ITSON), Ciudad Obregón, Mexico

In this article, the author transmits a very important message regarding means for bringing biodiversity into people's minds and government agendas (a rather difficult task) by connecting biodiversity to the everyday need for food coming from agriculture. The author presents a positive attitude by listing some examples of the environmental effects of agriculture and the tight connection with biodiversity, which succinctly underlines the need to rise a concern for biodiversity a planetary driver as we have done in the last two decades with the awareness of climate change.

I have read this submission. I believe that I have an appropriate level of expertise to confirm that it is of an acceptable scientific standard.

# Peer review: Referee response for version 1

**DOI:** 10.5256/f1000research.11119.r18238

**Published:** 2016-12-28

**Authors:** Mónica E. Riojas

**Affiliations:** 1 University Center for Biological and Agricultural Sciences (CUCBA), University of Guadalajara, Zapopan, Mexico

The manuscript addresses three avenues related with the relationship between biodiversity conservation and food production if we are meant to manage our natural resources in a reasonable way to assure the provision of environmental services in the future. These ideas are (1) the importance of the conservation of the ample genetic diversity of domesticated varieties of plants and their wild relatives; (2) the food acquisition habits of the population and, (3), in my opinion, the strongest idea, the integration of the ministries of agriculture and environment into one single entity. The three key ideas are important, but they are not developed to their full expression, and do not connect clearly with the central theme of the manuscript. I suggest that the author develops the two first ideas more comprehensively, and go more in depth in the third, so he can round up the message he wants to convey.

I have read this submission. I believe that I have an appropriate level of expertise to confirm that it is of an acceptable scientific standard.
